# Hybrid Fiber Optic Sensor Systems in Structural Health Monitoring in Aircraft Structures

**DOI:** 10.3390/ma13102249

**Published:** 2020-05-13

**Authors:** Karolina Bednarska, Piotr Sobotka, Tomasz Ryszard Woliński, Oliwia Zakręcka, Wiktor Pomianek, Agnieszka Nocoń, Piotr Lesiak

**Affiliations:** 1 Faculty of Physics, Warsaw University of Technology, Koszykowa 75, 00-662 Warszawa, Poland; karolina.bednarska.dokt@pw.edu.pl (K.B.); piotr.sobotka@pw.edu.pl (P.S.); tomasz.wolinski@pw.edu.pl (T.R.W.); 2 Polskie Zakłady Lotnicze Sp. z o.o., Wojska Polskiego 3, 39-300 Mielec, Poland; oliwia.zakrecka@lmco.com (O.Z.); wiktor.pomianek@lmco.com (W.P.); agnieszka.m.nocon@lmco.com (A.N.)

**Keywords:** aviation industry, hybrid optical fiber sensor, structural health monitoring, fiber Bragg grating sensors, polarimetric sensors, distributed sensors, optical time domain reflectometry

## Abstract

‘Smart’ structural health monitoring of composite materials with optical fiber sensors is becoming more and more important, especially in the aviation industry. This paper presents an overview of hybrid fiber-optic sensing systems based on scattering techniques, fiber Bragg gratings, interferometric techniques, and polarimetric methods in structural health monitoring. The main purpose of this manuscript is to analyze the possibilities of using hybrid sensors based on fiber optics to monitor composite structures, with a particular emphasis on aircraft structures. Since it is difficult to indicate the most comprehensive approach due to different parameters of the described sensors, the review contains a detailed description of available solutions. We hope that this work will allow for a better and faster selection of the right solution for the problem at hand.

## 1. Introduction

The aviation industry is a pioneer in attempts to implement the latest technologies in its constructions. The production of modern aircraft structures requires the use of materials with high mechanical strength while reducing the weight of structural elements. To meet the above requirements, modern materials are increasingly used, including composite materials, which are difficult to monitor their condition during impacts. This is due to the complex construction of composite parts. Each part consists of a specific number of layers, which in turn work differently depending on the orientation of the fibers and the forces acting. Additionally, aircraft structures are exposed to numerous failures and structural damages. The defects in the material microstructure, manufacturing errors, cutouts, holes, tight rounding radii and impacts of the structure are several reasons of failures. They can occur when an element or a structure is unable to withstand stresses that have been applied to it. That is why it is important to develop a technology that is able to monitor internally the structure state [1].

The aviation industry is much more demanding compared to, for example, telecommunications networks, where optical fibers are used as a standard. Based on conditions on board in civil and military aircrafts presented in [2], effects induced by large temperature changes, operating mechanical loads and shocks, mechanical vibrations as well crash safety should be taken into account. All these factors not only affect the selection of the right type of fiber-optic sensor, but also limit the choice of measuring equipment. 

However, optical fibers are being increasingly considered for use in aircrafts due to their numerous advantages over the existing electrical wires-based systems. Their most important feature is low weight, which may result in fuel consumption saving throughout the lifetime of the aircraft. Moreover, they are small in size and thus can be embedded into a structure without significantly affecting its physical properties. In addition, one optical fiber is much lighter and cheaper than the currently used electric cables. Thanks to the development of optical fiber techniques, it is possible to place multiple sensors on one optical fiber, which gives the opportunity to limit the number of optical fibers used, while maximizing the amount of information obtained. A smaller number of implanted fibers also means a smaller weight share in the aircraft structure and smaller impact on the aircraft [3]. Measurements done with optical fibers can be performed along the whole length of the sensor, both in localized and distributed mode and notably, they allow for simultaneous measurement of several physical quantities. Since optical fiber sensors are insensitive to electromagnetic fields, and can be used in applications where other types of sensors fail. 

The future of materials used in aviation constructions belongs to composites, among others due to their high strength-to-weight ratio. Thanks to that, designers can create lighter constructions than currently used metal consortia, which at the same time can withstand applied loads. The most important features of fiber reinforced composites in aerospace or construction applications are damage tolerance and damage resistance under applied loading due to the fact that they are exposed to numerous unplanned impact loads during the manufacturing process and in service.

**Figure 1:**
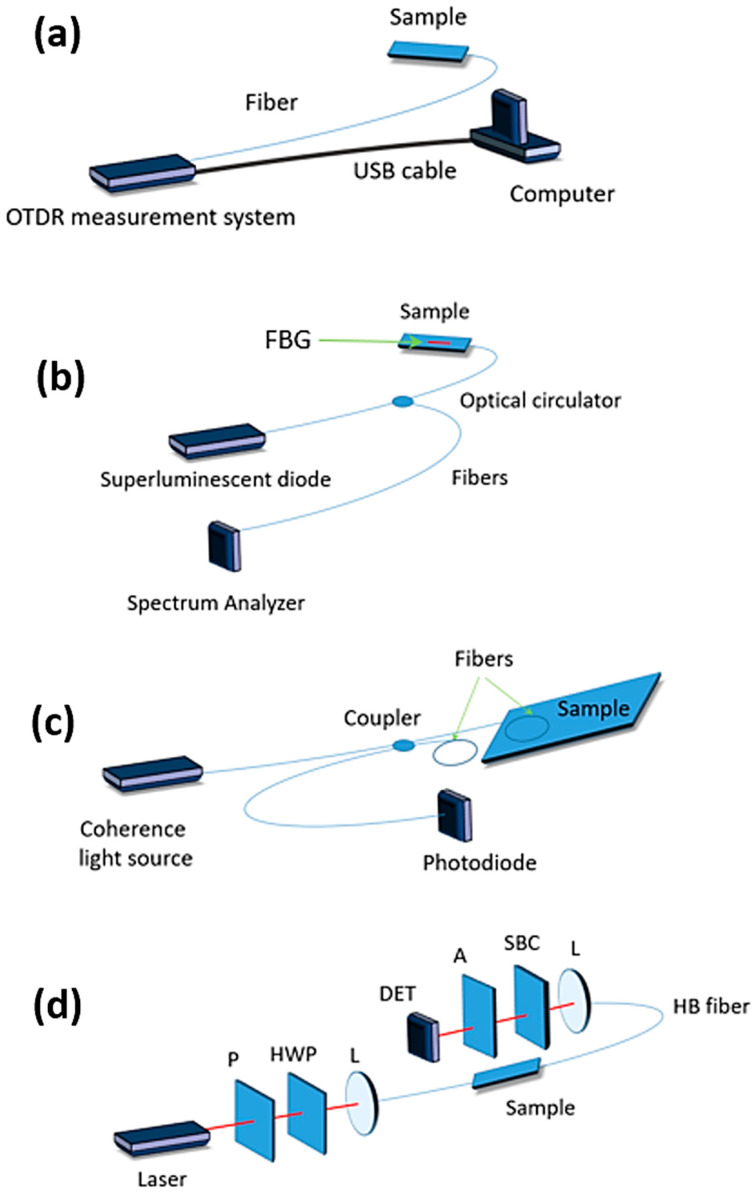
Scheme of classical sensors to be used for structural health monitoring (SHM) (**a**) scattering, (**b**) fiber Bragg grating, (**c**) interferometric and (**d**) polarimetric based on highly birefringent (HB) fibers, where: Polarizer (P), analyzer (A), half wave plate (HWP), Soleil-Babinet compensator (SBC), lens (L), detector (DET) are presented.

Structural health monitoring (SHM) has become an important process in maintaining the proper state of composite structures. One of the ways to execute it is by using approaches based on optical fibers, i.e., Raman or Brillouin scattering techniques [4,5,6,7], fiber Bragg gratings (FBGs) [8], interferometric [9] or polarimetric methods [10,11] (Figure 1). What makes optical fibers ideal for embedding in composites is the fact that silica glass optical fibers exactly match the properties of fiberglass-based composite materials.

Optical fibers can also offer a possibility to monitor the structure during its manufacturing process. Embedding such sensors into a fiber-reinforced epoxy composite allows for monitoring the structural health during the time of curing and general aging, helping to detect any potential defects and damages [12,13] as well controlling aging variations in resins from different batches. 

However, what makes fiber-optic sensors important for application in structural health monitoring is the possibility of creating a hybrid system that, in contrast to known conventional systems, is capable of multifunctional measurements. One such example is the ability of hybrid systems to perform static strain measurement in a large scale for potential operational load monitoring or ultrafast strain measurement in a small scale for potential damage detection. The main idea behind the development of hybrid systems is enhancing the positive aspects of conventional technology while, at the same time, minimizing its disadvantages. 

## 2. Hybrid Sensors Based on FBG 

**Figure 2:**
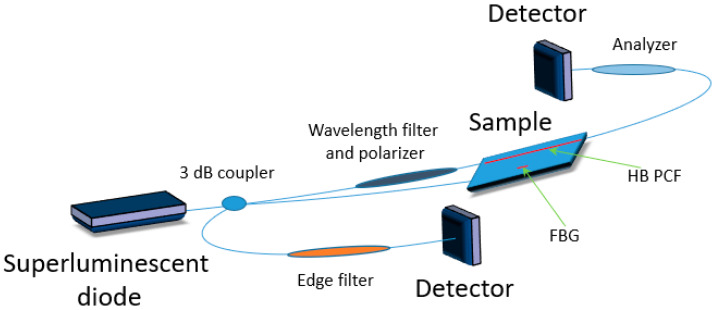
Scheme of a hybrid sensor based on a fiber Bragg grating (FBG) and highly birefringent polarization maintaining fibers where a highly birefringent photonic crystal fiber (HB PCF) is presented.

Currently, one of the most widely used technologies for implementing structural health monitoring is based on the use of FBGs. However, FBG sensors suffer from cross sensitivity between strain and temperature. A number of different methods have been proposed to overcome this simultaneous dependence, although their implementation is limited by the embedding of sensors in the host composite [14,15,16,17,18]. An answer to this issue may be the use of a hybrid sensor based on an FBG and highly birefringent (HB) polarization maintaining (PM) fibers. Such fibers can be made temperature insensitive and can allow for independent strain measurement. Typically, polarimetric sensors require a zero-strain reference, but a combination of HB fibers with FBG sensors enables to overcome their drawbacks. While HB fibers-based polarimetric sensors compensate for FBGs’ temperature sensitivity, the FBGs can provide zero strain reference needed for polarimetric sensors [19], thus creating a hybrid system for strain, temperature and vibration sensing (Figure 2). 

**Figure 3:**
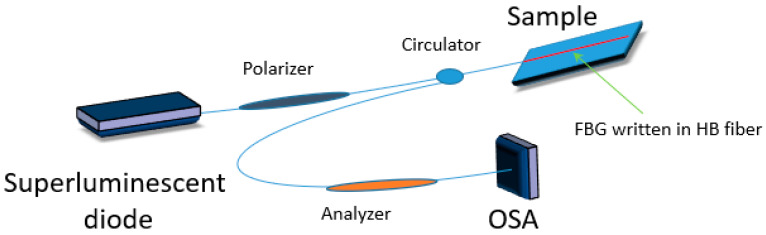
Scheme of hybrid sensor based on an FBG written in a highly birefringent polarization maintaining fiber.

Multiaxial strain measurement is required in some of the SHM applications for strain mapping. It has been reported elsewhere [20,21] that FBGs written in HB fibers have a real potential to simultaneously measure transverse strain and axial strain (Figure 3). Two Bragg peaks corresponding to both orthogonally polarized modes are displayed by the sensor and a change in the Bragg peak separation is dependent on the phase modal birefringence variation that is induced by transverse load and temperature. Specific properties of the sensor and its sensitivities to different values that are measured are determined by the type of used fiber. However, one of the biggest disadvantages of FBGs written in HB bow-tie fibers is greater temperature and strain cross sensitivity [22].

Multiple techniques can be used to realize simultaneous temperature and strain measurements. In [23] the authors present a fiber optic sensing system for real-time monitoring of the cure kinetics of glass fiber/epoxy composites. This system allows for in situ simultaneous measurement of both temperature and strain by the use of two coupled fiber Bragg gratings embedded into the composite.

In [24] the authors developed a hybrid grating system using the difference in strain and temperature response of fiber Bragg gratings and a long period fiber grating (LPFG) to discriminate between strain and temperature induced wavelength shifts. 

In [25,26] the authors proposed the use of hybrid FBG/EFPI (extrinsic Fabry-Perot interferometer) sensors for simultaneous temperature and strain measurements during the cure of composite laminates. While an FBG was used for temperature sensing, the EFPI sensor measured either mechanical or apparent strain, thus providing discrimination between temperature and strain induced signals. The overall accuracy of the system depends mostly on the exact dimension of the Fabry-Perot cavity. 

Most notably, the hybrid uses of FBG sensors and acoustic inspection for damage detection have recently become a trend in aircraft structural health monitoring. In recent years one of the most widely used systems has been based on embedding FBG sensors in laminates for the detection of ultrasonic waves travelling through them. This can be realized either in a passive (acoustic emission) or active mode. In the latter case, the sensors are combined with piezoelectric wafers that act as Lamb wave emitters [27] and the detection is based on tracking the varying pitch caused by propagating elastic waves [28]. The Lamb waves are sometimes called “plate waves” that propagate in a solid plate with free boundaries, both parallel and perpendicular to the plane of the plate. Such waves have been used ever more in several non-destructive testing (NDT) applications since their discovery by Lamb in 1917 [29] and their first NDT in 1957 [30]. The generation and detecting of Lamb waves can be realized by numerous techniques, but the most popular one is based on the use of piezoelectric actuators arranged in such a way that their axis is normal to the surface of the plate. Then, the received Lamb wave is reconstructed from the corresponding Bragg wavelength shift. 

**Figure 4:**
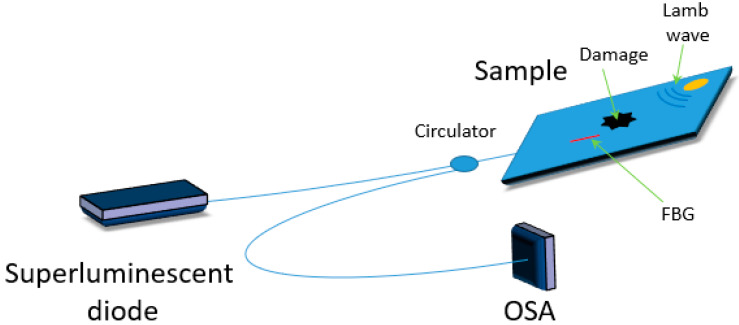
Scheme of hybrid sensor based on FBG and piezoelectric systems.

By joining such piezoelectric systems with FBG sensors it is possible to realize a hybrid system that optically monitors how the Lamb waves interact with structural defects (Figure 4). The use of such hybrid systems for structural health monitoring was reported by [31].

In [32] Qing et al. developed an active hybrid system that uses a bonded lead zirconate titanate (PZT) actuator and an FBG optical fiber sensor to monitor several types of damage, such as delamination and debonding. The piezoelectric transmits elastic waves into the structure, while the embedded FBG receives the corresponding structural response. 

**Figure 5:**
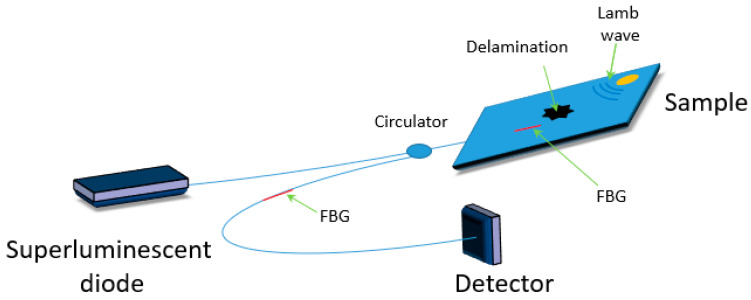
Scheme of a hybrid sensor based on two FBGs and piezoelectric systems.

Tsuda [33] proposed a system that uses a secondary FBG filter, cascaded with the FBG sensor (Figure 5). The first FBG was responsible for causing a phase shift in the reflected spectrum that intersected with the spectrum of the latter sensor. Such a system could detect a damage area of 65 mm × 15 mm. However, to achieve operation such architecture required the center wavelength of the FBG to be within a close range. In [34] Lam proposed a similar system, able to detect a delamination of 20 mm in glass fiber-reinforced epoxy.

Tan et al. proposed in [35] an ultrasonic excitation fiber grating sensing system, along with a location algorithm based on elliptic technology for damage detection. The detectable damage size threshold was improved to 6 mm. The estimation method of the algorithm was experimentally shown to be simple, however, the precision of location detection was limited by the change of ultrasonic wave mode. 

In recent years, Barazanchy et al. [36] have developed a hybrid system using a piezoelectric actuator and an FBG sensor to detect and localize damage located on a carbon fiber reinforced plastics aircraft plate skin. The system was able to detect thickness damage (a 2 mm diameter hole) as well as surface damage (a 2 mm diameter hole with a 0.65 mm depth). The detection of such small damage was possible due to the use of an elliptical triangulation algorithm [35,37]. Unlike previous hybrid SHM systems, this system used a lower number of sensors and allowed to detect damage in the area outside the sensor enclosure. This approach appears to be better suited for applications in large aerospace structure health monitoring.

**Figure 6:**
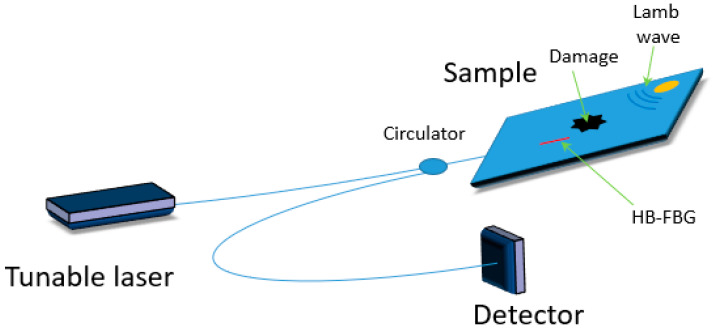
Scheme of a hybrid sensor based on FBG inscribed in an HB fiber and piezoelectric systems.

Rao and Duan presented in [38] Lamb wave detection by a combination of PZT actuators with FBG sensors inscribed in an HB fiber (Figure 6). Lamb waves launched from two angles (0 and 90°, relative to the sensor orientation) induced different responses in the sensor. The authors showed that through selective interrogation of the fast and/or slow axis of the constructed sensor it is possible to achieve bidirectional detection that allowed to differentiate the direction of Lamb waves propagation. Additionally, bimodal detection with an ability of single-mode sensing was shown to be possible. 

In Reference [39] Feng et al. presented a multi-parameter sensor that integrates 7-core FBGs, spatially multiplexed Raman distributed temperature sensor (RDTS) and polarization detection-based vibration sensor (PVS). Such a solution allows for vibration frequency and discriminated temperature and strain detection. The authors show that this system is capable of precisely measuring vibration frequencies, irrespectively from the applied strain and ambient temperature. The above described sensor was not laminated in a composite; however, this solution that embeds multiple independent sensors in a single, all-solid cladding may appear ideal for SHM due to its compactness.

## 3. Hybrid Interferometric Sensors

**Figure 7:**
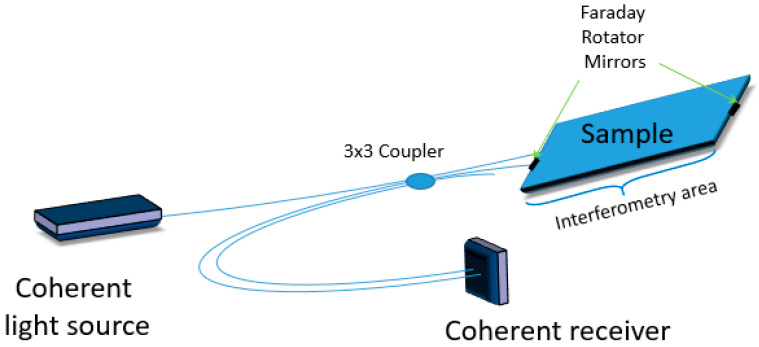
Scheme of a hybrid sensor based on coherent transmission in optical fibers.

**Figure 8:**
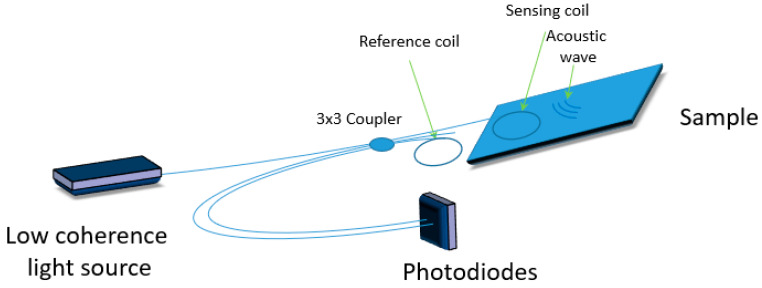
Scheme of a hybrid sensor based on combined sensing fiber coils.

Interferometric sensors operating in the hybrid regime are another type of systems used for SHM. In [40], an interferometric approach is used for the acquisition of dynamic signals for both active and passive impacts monitoring in metallic structures (Figure 7). It employs an innovative phase-diversity coherent detection scheme which retrieves the high-frequency phase information of received optical signals in a completely passive way. This is a novel solution based on optical fibers that can be configured in different manners to match the specified monitoring application. However, the use of this solution is usually a tradeoff between sensitivity and need for localized measurement, as e.g., in the acquisition of very high frequency dynamics. In this solution the authors have shown that although a large gauge length is necessary to achieve high sensitivity, there exists a threshold gauge length above which the signal becomes distorted. To guarantee local measurement, the sensing fiber is convoluted in multiple loops (in order to increase interferometric sensitivity) and glued by a short length to the structure. The first experimental verification of this solution was demonstrated by Djinovic et al. [41,42] for both passive acoustic emission monitoring and active damage monitoring (Figure 8). The authors combined sensing fiber coils with the interferometric approach and described how the system configuration (i.e., the gauge length and the number of optical loops) can be optimized to achieve maximized performances of the coherent fiber optic solution for active and passive monitoring applications. The strain waves were generated first by a piezoelectric transducer and then by an impulsive dynamometric hammer on an aluminum skin panel. Next, the signal was acquired by a fiber optic coherent sensor and verified both numerically and with traditional piezoelectric sensors, confirming a very good agreement. 

**Figure 9:**
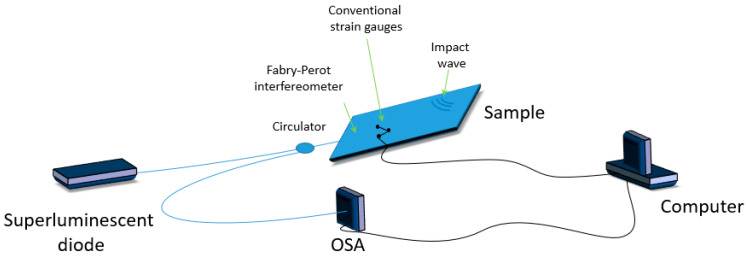
Scheme of a hybrid sensor based on Fabry-Perot interferometer and piezoelectric systems.

Akhavan et al. described in [43] the transient measurements of impact-induced strain in a graphite/epoxy composite by using surface-mounted extrinsic Fabry-Perot fiber optic sensors, conventional resistance strain gauges, and polyvinylidene fluoride (PVDF) piezo film sensor (Figure 9). Two sample plates made of eight-ply graphite/epoxy composite laminates were subjected to low-velocity impacts using a drop-weight tower. The plates were impacted with rigid spheres using the drop-weight technique. The authors noted that the impact events have not caused any damage to either plate. The obtained sensor strains were similar for each type of sensor, however, in terms of sensitivity and accuracy, the performance of the fiber optic strain sensor was superior to other strain gauges.

## 4. Polarimetric Sensors

Over the last decades much attention has been given to polarimetric optical fiber sensors based on highly birefringent polarization maintaining fibers [44,45]. Due to anisotropic stresses induced either by stress-applying elements placed along the length of the fiber or by designing noncircular core/cladding geometry (typically elliptical) this type of fiber has a built-in and well-defined internal high birefringence. In HB fibers the coupling between the phase velocities for the two orthogonally polarized modes is avoided owing to a sufficiently large effective refractive indices difference between these two eigenmodes.

**Figure 10:**
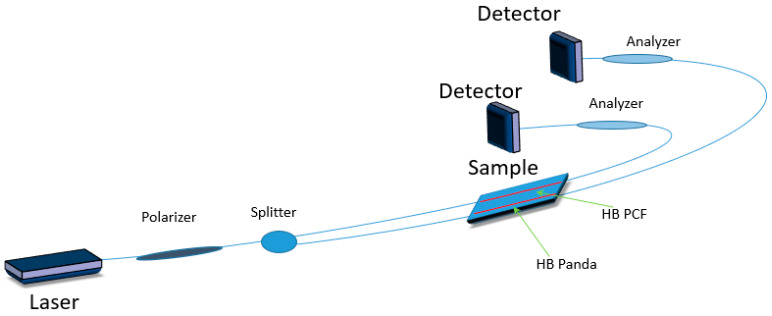
Scheme of a hybrid sensor based on two different highly birefringent fibers (side hole fiber and PCF).

In [46], a hybrid sensor was developed by combining two polarimetric sensors based on two types of HB fibers: A side-hole fiber and a photonic crystal fiber (PCF) (Figure 10). Both sensors are characterized by strain sensitivity, while the HB PCF sensor is temperature insensitive. Such a configuration allows independent strain measurement at ambient temperature. However, during the polymerization process the matrix material undergoes polymerization-based shrinkage, while the fiber remains volumetrically stable at the curing temperature. As a result of this mismatch, residual stresses are induced in laminated fibers, which is especially visible in highly birefringent fibers. 

During the polymerization shrinkage, the direction of displacement is perpendicular to the surface and associated with only one surface, i.e., the bonded polymer composite. When the reinforcement fibers are arranged parallel to the fiber axis, the displacement vectors are directed towards the center of the side surface of the fiber. In the case when the reinforcing fibers create a two-dimensional structure, the displacement vectors are asymmetric and directed mainly to the surface of the structure. As a result, the optical fiber embedded in the space between the two layers undergoes compression [47].

Different values of birefringence changes are obtained in the case of birefringent optical fibers laminated in a polymer structure in such a way that the reinforcing fibers create a two-dimensional structure. These changes can be either positive or negative, depending on the orientation of the fiber relative to generated stress [48]. 

## 5. Hybrid Sensors Based on Scattering Techniques

Optical time domain reflectometry (OTDR) has been widely used in communication and fiber sensing [49]. Rayleigh scattering based phase OTDR and Raman/Brillouin scattering based OTDR have been in development since the first demonstration of OTDR in 1976 [50]. Depending on the requirements of length and resolution of the measurand, different types of scattering can be used: Linear backscattering, non-linear backscattering and non-linear forward scattering. Raman scattering (for temperature) and Brillouin scattering (for strain and/or temperature) or their combination [51] using time, frequency, polarization, or correlation domain techniques (continuous or pulsed) including several variants [52], are used to interrogate the distributed transducer [53,54] (Figure 1c). One of the issues in such systems is the strain and temperature cross-sensitivity of the Brillouin frequency shift (BFS). In order to achieve simultaneous temperature and strain sensing in a single fiber, different techniques can be implemented, for example hybrid Raman-Brillouin scattering detection [51] or simultaneous detection of both Brillouin intensity and BFS [55]. Both of these techniques allow to distinguish temperature and strain. However, their applications are limited by several drawbacks. In the hybrid Raman-Brillouin detections systems, the measured strain-independent spontaneous anti-Stokes band is used both to correct the temperature dependence of BFS and estimate the fiber temperature. This scheme provides a reliable, temperature-independent strain measurement [56]. The advantage of distributed sensing over multiplexed point sensing is that spatially continuous measurement without dark zones can be achieved.

Bolognini et al. [57] used standard Fabry-Perot (FP) lasers to achieve simultaneous distributed temperature and strain sensing based on the hybrid Raman-Brillouin technique. It appeared that FP sources can be used to obtain enhanced hybrid sensing systems due to their inherently multi-longitudinal modes. The authors improved the sensing performance by exploiting a multi-wavelength oscillator and a single photon-detector for the simultaneous measurement of the BFS for all FP modes. The use of the FP lasers allowed to overcome the issues related to the coherent Rayleigh noise (due to the large FWHM) and provided a significant rise in accuracy for both temperature and strain.

In [58] Zhang et al. demonstrated a distributed fiber sensing system for multi-parameter detection of vibration, temperature and strain. This was achieved by integrating phase sensitive OTDR (φ-OTDR) and Brillouin OTDR (B-OTDR). The three measured parameters can be extracted simultaneously by only one photodetector and one data acquisition channel, since the vibration is fast changing while temperature and strain have static properties. Owing to this, both the width and intensity of laser pulses are modulated and injected into a single mode sensing fiber proportionally. 

Taki et al. [59] demonstrated the use of cyclic pulse coding for a hybrid Raman/Brillouin optical time-domain analysis distributed sensing system. The proposed technique allows for temperature and strain measurement implementation with a meter-scale spatial resolution over 10 km of standard single mode fiber.

In Reference [60] Muanenda et al. designed and demonstrated a hybrid distributed fiber-optic acoustic and temperature sensing system through integrating Raman distributed temperature sensors (RDTS) and Rayleigh φ-OTDR techniques. A linear pulse coding scheme is implemented to ensure inter-pulse coherence and intra-pulse incoherence to achieve a high signal to noise ratio, thus increasing spatial resolution and sensing range. 

Basing on the integration of RDTS and φ-OTDR, Muanenda et al. [61] presented a hybrid distributed acoustic and temperature sensor. The authors used an off-the-shelf distributed feedback laser to control and modulate the optical source. This ensured that both the inter-pulse incoherence and intra-pulse coherence were achieved, which in turn enabled cyclic simple coding to be effective for both RDTS and φ-OTDR. In this way, accurate long-distance measurements of temperature and vibrations were achieved, requiring only a minimal amount of post processing, which is impossible in the case of static strain measuring. 

**Figure 11:**
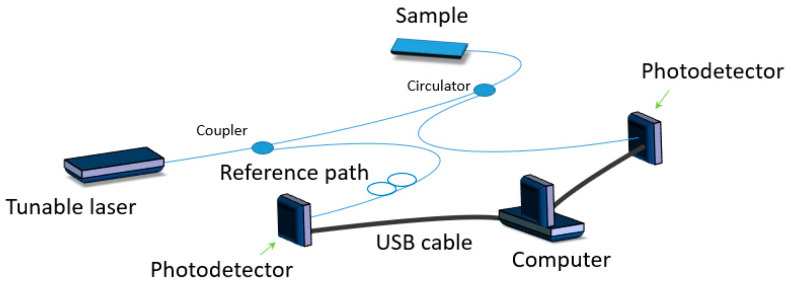
Scheme of a hybrid Brillouin–Rayleigh optical fiber sensing system based on reference path and a signal path.

In Reference [62], Ito et al. developed a hybrid Brillouin-Rayleigh optical fiber sensing system to monitor composite curing (Figure 11). A single optical fiber was embedded in a carbon fiber reinforced plastics laminate to independently measure temperature change and residual strain during the cooling period of the curing process. The system was able to identify a nonuniform thermal residual strain field induced by a nonuniform cure temperature. The measured values agreed well with those measured by a conventional system (i.e., fiber Bragg grating sensors and thermocouples). 

Jothibasu et al. [63] presented a distributed fiber optic strain sensor based on Rayleigh backscattering that was embedded between the layers of composite laminates to monitor, by using OTDR, the spatially continuous profile of strain. Tensile loads were applied using an Instron testing system and were successfully investigated with a distributed sensor embedded inside the composite samples. The results showed a linear response, which indicated seamless integration of the fiber in the composite material. 

## 6. Constraints Imposed by Composites on Aircraft

While designing an SHM system, several limitations that composite structures impose have to be taken into account. Below we point out some of these limitations, in relation to the use of composites in aircrafts.

(1) Weight sensitivity: The system must add a minimum amount of weight. A life-cycle calculation can be made to compare the obligatory replacement of a component after a certain number of flight hours with the addition of an instrumented component which has higher weight and cost.
(2) Thickness of structure: In most cases the thickness ranges between 0.5 to 3 mm (there are portions as thick as 10 or 20 mm, but very few).
(3) Aerodynamics: Nothing can be attached on the outside because it adversely affects aerodynamic performance and is subject to damage (e.g., hail damage, tool drops, runway debris, etc.)
(4) Access: If the system must be accessed for service or other reasons, it can only be used at limited areas where access doors are already available for maintenance or inspection. Adding extra access doors adds to the cost and weight. At the same time, typical areas of concern are at joints and 3-D structural details which are typically deep inside the structure. Thus, access is limited, which means the system must be robust, redundant and must have a long life.
(5) Entry-exit points of sensors: Given that sensors must be embedded, robust, and low cost, the entry and exit points must be used. Additionally, these must be low weight.
(6) Ply thickness: Typical composite plies are 0.1–0.3 mm thick. If the sensors are of the same size, then they either locally disrupt the strain field they are trying to measure or may act as stress concentrations leading to local failure.
(7) Environment: The system must withstand temperatures ranging from −50 to +75 °C, dry to fully saturated by humidity environments, and potential exposure to hydraulic fluids or jet fuel.

Table 1 summarizes all publications regarding hybrid sensors analyzed in the manuscript. The designation +/− in columns 1–7 determines whether the given solution meets the condition specified in the points 1–7 described earlier. The ‘Mode’ column denotes which parameters are measured by a given sensor; ‘+’ means that they are measured simultaneously, while the comma (,) signifies that measurements are not made at the same time.

By analyzing Table 1 it can be concluded that it is difficult to fulfill conditions 2 and 6 (due to a need to install the sensor in the composite structure) as well conditions 1 and 7 due to the expensive and sensitive to temperature changes and vibrations detection systems meeting the requirements described in [3]. The thickness of a single layer of the composite and the thickness of the structure for some solutions create a very big challenge. If, in addition, too much interference in the structure of the composite causes external changes in the shape of the composite material, it can badly affect the aerodynamic properties of the structure. It is particularly difficult to meet these excessive requirements with solutions based on interferometric sensors. They require special mounting of the sensor, installation of additional elements such as mirrors or installation of additional reference lines in which the measured interference will not occur. The second group of sensors that do not meet condition 3 are those that use a reference sensor placed under similar conditions but not in the composite structure. It is difficult to predict where the reference sensor will eventually be placed in such a solution, however, for the purpose of this analysis, we assumed that such a sensor does not meet the condition of not affecting the aerodynamics (3) or the conditions of placing the sensor in the composite material (2 and 6).

On the other hand, solutions based on scattering techniques are easier to be implemented in aircraft constructions due to the possibility of stress and temperature measurements inside a composite material by laminating only a single optical fiber. Depending on the expected resolution of the measurement, different length of the optical fiber placed in the composite material may be considered. Thanks to this, it is possible to monitor the entire elements (skins or spars) exposed to damage depending on the needs [48]. Unfortunately, the measuring apparatus used is extremely sensitive to the influence of environment and vibrations. Additionally, it is characterized by quite a significant mass, so these techniques do not meet the guidelines described in points 1 and 7. In the absence of a detailed description in the cited works, it was assumed that this kind of apparatus is used, which eliminates such a solution for aviation applications. However, if modifications that eliminate sensitive apparatus are used, such a solution can be used in aviation.

Relatively, the easiest approach is to use solutions based on highly birefringent optical fibers and FBGs. These solutions allow to measure stress and temperature inside a composite material by laminating a single optical fiber. A significant disadvantage of HB fiber-based solutions is non-linearity of the output signal requiring complex and expensive systems to be used to detect larger signals and high temperature sensitivity. Even small birefringence drifts due to temperature fluctuations can degrade strain sensitivity. 

Solutions based on FBG sensors are also sensitive to temperature changes. Hence, FBG sensors are characterized by their dual sensitivity to strain and temperature. For a typical aerospace operating temperature of 150 °C thermal effects contribute an additional apparent strain of up to 1000 με. On the other hand, a detection system using an interrogator is not very energy-consuming, being characterized by good resistance to vibrations and temperature changes [64,65]. A typical interrogator, thanks to the use of fit techniques to achieve a wavelength fit resolution of up to 0.3 pm and frequency of 500 kHz, allows real-time spectrum monitoring [66,67]. (The only disadvantage of the device (along with the broadband SLED light source) is their “volumetric” nature, based on bulk elements such as diffraction gratings, detector lines, optical lenses, etc. One of the solutions to this problem are photonic integrated circuits (PIC) based interrogators [68,69].

Given the above constraints, the most promising systems dedicated for SHM are those using FBGs and hybrid solutions combining the advantages of Rayleigh and Brillouin sensors both using lightweight and resistant to temperature changes and vibrations detection systems. The list of all hybrid fiber optic sensor systems discussed in the manuscript indicates that several of the proposed solutions [19,20,21,23,38,39,46,57,58,60,61] allow simple application in civil and military aircraft. However, FBG sensors deserve special attention. Using appropriate lamination techniques, FBGs can be safely placed in a selected location to monitor temperature or deformation in both static and dynamic modes.

## 7. Conclusions

To summarize, in this review we analyzed different possibilities of using hybrid fiber optic sensor systems embedded in composite materials as effective sensors of various physical quantities, including temperature, strain, stress, vibrations, and mechanical defects. Depending on specific applications, the hybrid sensors, both in point and distributed, can operate either in reflection or transmission regimes measuring simple or complex stresses. The main idea behind developing hybrid systems is enhancing the positive aspects of conventional technology, while at the same time minimizing its disadvantages.

Composite materials are increasingly used in innovative construction of both commercial and military aircraft. They also gained popularity in modern unmanned aircraft. Unfortunately, due to the possibility of barely visible damages occurring on their outer surfaces, it is necessary to monitor the structural health of the most vulnerable structures. Monitoring techniques using optical fibers allow the monitoring of the structure from the inside. This is possible due to the embedding of optical fibers into the interior of a composite material. Hybrid optical fiber sensors embedded in composite materials that show the ability to discriminate between temperature, strain, and thermal strain have great potential for novel fiber optic based SHM applications. 

## Figures and Tables

**Table 1:** Comparison of the properties of the presented hybrid measurement techniques.

	Ref.	1	2	3	4	5	6	7	Mode	Remarks
FBG based	19	+	+	+	+	+	+	+	Strain + temperature (static mode)	Strain sensitivity 0.0012 dB/με, temperature sensitivity 0.0039 dB/°C
	20	+	+	+	+	+	+	+	Temperature, strain (transversal + axial) (static mode)	Axial strain sensitivity 1.12 pm/me, transversal strain sensitivity 0.26 pm/mε, temperature sensitivity −0.42 pm/°C
	21	+	+	+	+	+	+	+	Temperature + strain (static mode)	Strain sensitivity up to 1.22 pm/mε, temperature sensitivity 4.41 pm/°C
	23	+	+	+	+	+	+	+	Temperature + strain (dynamic and static mode)	Dynamic loads up to 10 Hz, strain sensitivity 7.87 mm^−1^, temperature sensitivity 6.14 °C^−1^
	24	−	+	+	+	+	+	+	Temperature + strain (static mode)	Strain accuracy 5 mε, temperature accuracy 0.5 °C
	25	+	−	−	+	−	−	+	Temperature + strain (static mode)	Strain accuracy 20 mε, temperature accuracy 11 °C
	26	+	−	+	+	+	−	+	Temperature + strain (static mode)	Strain sensitivity 47.68 mε/nm, temperature sensitivity 97.13 °C/nm
	31	−	−	−	+	+	−	−	Damage detection (dynamic mode)	Detection up to 300 kHz of the acoustic wave, damage localization up to 50 mm from the sensor
	32	−	−	−	+	+	−	−	Damage detection (dynamic mode)	Detection up to 2 kHz of the acoustic wave
	33	−	−	−	+	+	−	−	Damage detection (dynamic mode)	Detection up to 100 MHz of the acoustic wave
	34	−	−	−	+	+	−	−	Damage detection (dynamic mode)	Detection up to 1.3 MHz of the acoustic wave,
	35	−	−	−	+	+	−	−	Damage detection, localization, (dynamic mode)	Detection up to 1 MHz of the acoustic wave
	36	−	−	−	+	+	−	−	Damage detection + localization (dynamic mode)	Detection up to 400 kHz of the acoustic wave, damage localization up to 25 mm from the sensor
	38	+	+	+	+	+	+	+	Bidirectional, Lamb wave (dynamic mode)	Frequency range up to 140 kHz
	39	+	+	+	+	+	+	+	Multicore, vibration + strain + temperature (dynamic and static mode)	Temperature sensitivity 19.2 pm/°C, strain sensitivity 1.2 pm/m
Interferometric techniques	40	+	−	−	+	+	−	+	Strain (dynamic mode)	Reference line, frequency range up to 1.5 kHz
	41	+	−	−	+	+	−	+	Damage detection (dynamic mode)	Reference line, frequency range up to 200 kHz
	42	+	−	−	+	+	−	+	Damage detection (dynamic mode)	Reference line, frequency range up to 300 kHz
	43	+	−	−	+	+	−	+	Damage detection (strain) (dynamic mode)	Frequency range up to 500 MHz
HB based	46	+	+	+	+	+	+	+	Strain, temperature (static mode)	Strain sensitivity is equal to 4 rad/m*mε
Scattering techniques	57	+	+	+	+	+	+	+	Temperature + strain (static mode)	Strain (temperature) resolution of 100 με (1.2 °C) at 25 km distance
	58	+	+	+	+	+	+	+	Vibration, strain, temperature (dynamic mode)	Detection up to 4.8 kHz of the vibrations, strain coefficient 0.0495 MHz/με, temperature coefficient ~0.9876 MHz/°C
	59	+	+	−	+	+	+	+	Temperature + strain (static mode)	Reference line, strain resolution 80 mε, temperature resolution 3.4 °C,
	60	+	+	+	+	+	+	+	Vibration + temperature (dynamic mode)	Frequency range up to 500 Hz, temperature sensitivity 0.5 °C, spatial resolution—5 m
	61	+	+	+	+	+	+	+	Vibration, temperature (dynamic mode)	Frequency range up to 500 Hz, temperature sensitivity 0.5 °C, spatial resolution—5 m
	62	−	+	+	+	+	+	−	Strain, temperature (static mode)	Strain coefficient Brillouin scattering C1 = 5.077 × 10^−5^ GHz/μɛ and Rayleigh scattering, D1 = −1.477 × 10^−1^ GHz/μɛ; temperature coefficient of Brillouin scatterings C2 = 1.090 × 10^−3^ GHz/°C and Rayleigh scattering, D2 = −1.498 GHz/°C.
	63	+	+	−	+	+	+	+	Strain (static mode)	Reference line, 1 mm spatial strain resolution
